# Feasibility of a novel self-collection method for blood samples and its acceptability for future home-based PrEP monitoring

**DOI:** 10.1186/s12879-022-07432-0

**Published:** 2022-05-13

**Authors:** Chase A. Cannon, Meena S. Ramchandani, Matthew R. Golden

**Affiliations:** 1 Department of Medicine, University of Washington, 325 9th Ave, Box 359777, Seattle, WA USA; 2 HIV/STD Program, Public Health-Seattle & King County, 325 9th Ave, Box 359777, Seattle, WA USA

**Keywords:** PrEP, Home sampling, Blood self-collection, Syphilis

## Abstract

**Background:**

Most non-clinic based HIV pre-exposure prophylaxis (PrEP) programs require fingersticks to self-collect blood specimens for laboratory monitoring, a technique that often results in inadequate blood volume for quantitative syphilis and HIV serological testing. We evaluated the acceptability and feasibility of using the Tasso OnDemand™ device as a self-sampling method for PrEP monitoring tests and compared results from samples obtained using the Tasso device to clinician-collected blood samples.

**Methods:**

We enrolled study subjects online and in a sexual health clinic and HIV clinic in Seattle, WA, USA to assess the acceptability of blood self-sampling and preferences for home-based PrEP monitoring. We compared HIV antigen/antibody, quantitative rapid plasma reagin and creatinine results in paired self-collected and clinical specimens collected from a subset of participants.

**Results:**

Of 141 participants, 124 (88%) were interested in collecting samples for PrEP monitoring at home. Among 48 who completed blood collections, 94% found the Tasso device easy to use and 95% felt they could perform self-sampling at home. Of 27 participants who used two devices, 100% collected sufficient blood to perform up to two tests while 33% collected sufficient serum for three tests. Agreement in test results between paired samples was high.

**Conclusions:**

These pilot data suggest that using the Tasso self-collection device is acceptable and could feasibly be used to obtain serum specimens sufficient for guideline-recommended PrEP monitoring, though use of a larger volume device may be preferable.

**Supplementary Information:**

The online version contains supplementary material available at 10.1186/s12879-022-07432-0.

## Background

Pre-exposure prophylaxis (PrEP) scale-up efforts in the United States (USA) have been hindered by the limited capacity of health systems to monitor rising numbers of PrEP users, lack of access to PrEP providers, and the burden of frequent, in-person monitoring visits that contribute to disengagement from PrEP care [1–3]. For key populations such as sexual minority men and transgender women, PrEP discontinuation is associated with future HIV acquisition [3], highlighting the need for targeted strategies to provide people with more options to continue PrEP. Home-based sample collection for HIV and sexually transmitted infections (STI) is an appealing, and often preferred, alternative to clinic-based testing [4, 5] that has the potential to minimize PrEP discontinuation by allowing additional flexibility for PrEP monitoring.

US Centers for Disease Control and Prevention guidelines recommend PrEP users be screened for STI every three months, including testing for syphilis and HIV [6]. To date, home-based PrEP (HB-PrEP) monitoring models have required persons to perform fingersticks at home to collect blood for PrEP laboratory testing [7, 8]. However, fingerstick sampling can be painful and typically does not produce a sufficient volume of blood to perform 4th generation HIV, creatinine, and nontreponemal serologic testing for syphilis. Given that many people on PrEP have a history of syphilis and consequently have positive treponemal test results in the absence of active syphilis, nontreponemal serologic tests (such as the quantitative rapid plasma reagin [RPR]) are necessary to identify persons who require syphilis treatment.

The Tasso OnDemand™ [9] (hereafter called Tasso) is a novel collection system designed to collect 250–300 µl of whole capillary blood from the upper arm using gravity and light suction without the need for venipuncture. The Tasso sampling system is registered as a USA Food and Drug Administration class 1 exempt device, similar to a medical lancet, and its use has been studied in several clinical and non-clinical settings [10, 11], including a recent study that measured SARS-CoV-2 antibody levels in study subjects who self-collected specimens unsupervised in a clinic [12]. At least two laboratories in the United States have also performed validations of creatinine testing using Tasso-collected blood specimens. However, the device has not yet been used in the context of monitoring individuals taking PrEP. In this paper we present results of a pilot study designed to assess the acceptability and feasibility of using the Tasso device for HB-PrEP. We also compared HIV, creatinine, and syphilis test results obtained using self-collected and clinician-collected serum samples.

## Methods

The study included two components. The first was a survey evaluating the acceptability of HB-PrEP and use of the Tasso device for specimen collection. The second involved having a subset of participants self-collect blood specimens using the Tasso device and answer questions regarding their experience. We also compared RPR, HIV antigen/antibody, and creatinine test results from clinician-collected venipuncture and self-collected Tasso specimens.

### Setting

Participants were patients at either the Public Health-Seattle & King County (PHSKC) Sexual Health Clinic (SHC) or the Max Clinic, both of which are located on the Harborview Medical Center campus in Seattle, WA, USA. The SHC provides comprehensive medical services related to STIs and is the largest single provider of PrEP in Washington State, with approximately 650 current PrEP patients. The Max Clinic is a walk-in clinic for persons living with HIV [13].

### Recruitment and enrollment

The study population comprised two groups enrolled from May 2020 to February 2021. The first group was recruited online from a subset of current or eligible PrEP users (approximately 75% of all SHC PrEP patients) who had agreed to receive communication from clinic staff through a secure text messaging platform. At study initiation and three months following initiation, we sent patients a recruitment text that contained a brief introduction to the study and a link to complete an online consent form.

The second group included English-speaking patients receiving in-person clinical services at the Max Clinic or SHC who had venipuncture specimens taken as part of their routine care. Additionally, patients had to be either eligible for or currently using PrEP, have a known or suspected new syphilis infection, or be living with HIV. Since our study began shortly after COVID-19 restrictions significantly reduced the number of face-to-face visits allowed at University of Washington (UW) and PHSKC facilities, the SHC population was comprised primarily of persons with symptoms suggestive of STI. Inclusion criteria for this second group were designed to ensure that the study included persons with positive HIV and syphilis tests to allow for evaluation of the agreement between venipuncture and Tasso device-collected specimens. Persons taking anticoagulant medications were excluded from performing the blood self-collection. Online consent forms and survey responses were collected and managed using secure, web-based REDCap (Research Electronic Data Capture) tools [14] hosted at the UW’s Institute of Translational Health Sciences. Survey instruments for both groups are available as appendices (Additional files 1, 2). Study procedures were approved by the University of Washington’s Human Subjects Division and institutional review board (study #00009004) and Public Health-Seattle & King County’s Research Administrative Review Committee (study #20-680).

### Procedures

#### Online group

After enrollment, participants were asked to view a brief video introducing the Tasso device and concept of remote PrEP monitoring and then complete an online survey on their interest in HB-PrEP. Online participants did not receive compensation for study participation.

#### In-person group

Eligible persons were referred to the study by medical providers after completing their clinic visit. Following enrollment, participants watched a training video [15] and received detailed instructions on how to obtain blood samples using the Tasso device (Additional file 3: Fig. S3). To simulate unsupervised home collection conditions and comply with UW COVID-19 distancing restrictions, study staff were not physically present during the blood self-collections but remained available as needed for assistance via telephone from offices adjoining the clinic rooms. The study ordered serum tests on self-collected specimens to match tests ordered during the clinical visit, allowing for paired comparisons between the clinician-collected venipuncture (gold standard) and self-collected Tasso samples. As such, the number of tests performed for each participant varied (e.g., a person attending a routine month 15 PrEP monitoring visit would typically have HIV and syphilis testing but not a creatinine test). In the first months after study initiation, we discovered that use of only one Tasso device often resulted in inadequate blood volumes to run all three tests. Thus, we revised study procedures to direct all participants to use two devices for sequential collections on either the same or different arms. After specimen collection, participants completed a survey on the acceptability of self-collecting specimens and likelihood of participating in a HB-PrEP program. In cases where collection was unsuccessful, we attempted to determine the likely cause by reviewing the collection technique and asking questions about recent activity in the day leading up to collection. All enrollees who attempted the blood collection and completed a post-procedure survey were offered a $10 gift card.

### Outcome and sample size considerations

We defined thresholds for acceptability and feasibility prior to initiation of the study. Acceptability was defined as ≥ 85% of participants reporting that the device was not difficult to use and that they could envision being able to self-collect a specimen at home. We defined feasibility as ≥ 80% of participants successfully self-collecting samples, without direct supervision, of volume sufficient to perform at least one of the three serum tests for PrEP monitoring.

Given the significant clinical implications of misdiagnosing HIV, we determined a priori that paired HIV specimens should be 100% concordant. For syphilis test accuracy, we specified that qualitative results would be concordant for ≥ 75% of cases and that paired quantitative titers would be different by ≤ 1 dilution, based on the fact that a two-titer difference is usually defined to represent a clinically significant change [16]. We defined the acceptable range of variation for creatinine specimens to be ± 0.15 mg/dL. Based on data from our SHC PrEP cohorts and previous evaluations of capillary vs. venous creatinine values that suggest venous sample results are on average 0.15 mg/dL higher [17], we hypothesized that the average creatinine estimates would be 1.1 and 0.95 mg/dL for venipuncture and Tasso specimens, respectively. Thus, we determined 10 or more paired samples would yield at least 80% power to evaluate whether the mean creatinine difference between specimens was within a clinically acceptable range.

### Analyses

All sample processing and testing was performed in Clinical Laboratory Improvement Amendments-certified facilities. Clinician-collected venipuncture samples were tested for HIV, creatinine and syphilis according to routine SHC protocols. Tasso-collected whole blood specimens were first delivered to the PHSKC laboratory for centrifugation, after which serum was used for testing. Sera were tested for HIV using the 4th generation GS HIV Combo antigen/antibody enzyme-linked immunosorbent assay (EIA) (Bio-Rad, Hercules, CA, USA) and for syphilis using the ASI RPR card (Arlington Scientific Inc. Springville, UT, USA). All positive RPR tests from Tasso samples were quantified up to a 1:16 titer; values beyond this were determined if residual specimen volume permitted. The CAPTIA™ syphilis IgG EIA (Bio-Rad, Hercules, CA, USA) was used for confirmation of discordant venipuncture/Tasso RPR specimens. HIV and syphilis tests were internally validated by the PHSKC laboratory prior to this study. Creatinine testing was performed at the UW laboratory using the automated Beckman Coulter AU analyzer (Beckman Coulter Inc., Brea, CA, USA).

We used descriptive statistics to characterize participant demographics, survey responses, and the proportion of participants who attempted and completed self-collections. For HIV and qualitative RPR values, we determined the percent positive agreement (PPA) and negative percent agreement (NPA) between venipuncture and self-collected specimen results. We also used descriptive statistics to characterize the means, standard deviations and differences for paired quantitative RPR titers and creatinine values. Correlation between the paired sample results was analyzed using Pearson’s correlation coefficients and linear regression. Quantitative RPR titers were plotted on the binary logarithm scale (e.g., value of 3 represents a titer of 1:8). All analyses were conducted using R version 3.5.1 (R Foundation for Statistical Computing, 2018) and used a two-sided alpha level of 0.05 for statistical significance.

## Results

Of 509 persons who were sent text messages offering them study enrollment, 118 (23%) completed the online survey. Forty-eight (84%) of 57 participants who were approached in the clinic to complete in-person procedures chose to enroll. Table 1 describes the demographics and behavioral characteristics of study participants and their survey responses. Most participants (90%) were cisgender men with a median age of 34 years; 81% were currently using PrEP. Over half (53%) of enrollees identified their race/ethnicity as non-Hispanic White, 15% as Latinx, and 10% as mixed or another race. More than a third of participants reported a history of syphilis. Over 80% of participants preferred HB-PrEP to clinic-based PrEP in the context of active COVID-19 pandemic restrictions, while 72–83% preferred home-based services in the absence of such restrictions. Almost half (45%) indicated that they would be more likely to stay on PrEP if they could collect specimens at home as part of HB-PrEP monitoring.

**Table 1:** Demographic characteristics and home-based PrEP (HB-PrEP) interest survey responses for in-person and online cohorts

	In-person (n = 48)^a^		Online (n = 118)^a^	
	n	%	n	%
Response type				
Demographic and behavioral				
Age (years), median (IQR)	33 (27–39)		35 (29–41)	
Gender				
Cisgender man	44	91.7	90	89.1
Non-binary	1	0.02	3	3.0
Other not listed	1	0.02	8	7.9
Race/ethnicity				
Non-Hispanic white	26	54.2	53	52.0
Latinx	6	12.5	17	16.7
Mixed race/other	4	8.3	14	13.7
Non-Hispanic black	4	8.3	9	8.8
Gender of sex partners^b^				
Cisgender men	43	89.6	94	79.6
Cisgender women	5	10.4	7	5.9
Transgender man/woman	3	6.3	20	16.9
Non-binary/other not listed	8	16.7	12	10.2
Currently on PrEP	34	70.8	101	99
Duration of PrEP use (years), median (IQR)	2.5 (1.6–3.9)		2.5 (1.5–4)	
History of prior syphilis	16	33.3	21	39.6
Survey Items				
Given current COVID restrictions, are you interested in collecting your own PrEP lab samples at home?				
Yes	34	87.2	90	88.2
Maybe/not sure	5	12.8	8	7.8
No	–	–	4	3.9
When COVID restrictions end, would you be interested in HB-PrEP over coming to clinic?				
Yes	24	82.8	74	72.3
Maybe	5	17.2	21	20.5
No	–	–	5	4.9
If home testing required you to collect blood in a small tube at home, how would you prefer to collect specimens?				
Come to clinic for blood draw	7	17.9	24	24.5
Collect blood sample at home	32	82.1	74	75.5
Would the option to mail in kits from home make you more likely to stay on PrEP?				
More likely	15	38.5	46	46.9
About the same	22	56.4	50	51.0
If we could not perform syphilis testing, would you still be interested in home kits for HIV, gonorrhea, chlamydia testing only?				
Yes	34	87.2		
Maybe/not sure	4	10.3		
Did you find the process to collect blood using the Tasso device was difficult?				
Yes	0	0.0		
No	45	93.8		
Somewhat	3	6.3		
Do you feel like you could collect a blood sample like this on your own at home?				
Yes	37	94.9		
Maybe/not sure	2	5.1		
What are you concerns about collecting your own blood sample at home?				
None	45	93.8		
Identified concern	3	6.2		

*HB-PrEP* home-based PrEP, *IQR* interquartile range, *PrEP* pre-exposure prophylaxis

^a^Sums and percentages may not add to total or 100%. All questions were optional and response counts varied

^b^Participants could select more than one gender

**Fig. 1:**
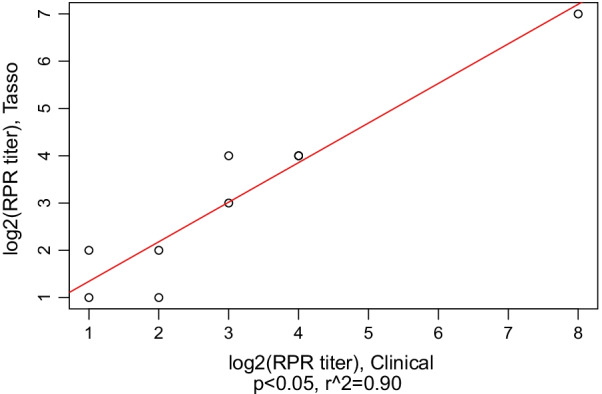
Comparison of qualitative RPR values from self-collected Tasso vs. clinical venipuncture specimens

Thirty-six specimens were tested for HIV: PPA and NPA between clinical and self-collected specimens were both 100%. Similarly, PPA was 100% for 10 reactive RPR specimens. Counts for paired qualitative HIV and RPR results are presented in Additional file 4: Table S1 and Additional file 5: Table S2. Four (40%) of 10 specimens had different RPR titers between self-collected and venipuncture specimens, but in all instances these differences were within one dilution, with half being higher using self-collected specimens and half being lower (Table 2). The quantity of blood was insufficient to perform titers beyond 1:16 for one case (Participant I). Correlation between self-collected and venipuncture specimen RPR titers was high (Fig. 1; r^2^ = 0.90, *P* = 0.0001). NPA was 95% for 21 paired RPR specimen results. Confirmatory syphilis EIA testing of the single discordant pair of RPR results was negative, likely consistent with an initial false positive venipuncture specimen.

**Table 2:** Comparison of quantitative RPR titer results for paired clinician-collected venipuncture and self-collected Tasso samples

Participants with positive qualitative RPR	Venipuncture sample result	Tasso sample result	Dilution difference between venipuncture and Tasso samples
A	1:256	1:128	+ 1
B	1:16	1:16	0
C	1:8	1:8	0
D	1:16	1:16	0
E	1:4	1:2	+ 1
F	1:4	1:4	0
G	1:2	1:4	− 1
H	1:2	1:2	0
I	1:512	> 1:16^a^	N/A
J	1:8	1:16	− 1
			Mean difference = 0 dilutions

*N/A* unable to calculate

^a^Insufficient sample to quantitate titer beyond 1:16

**Fig. 2:**
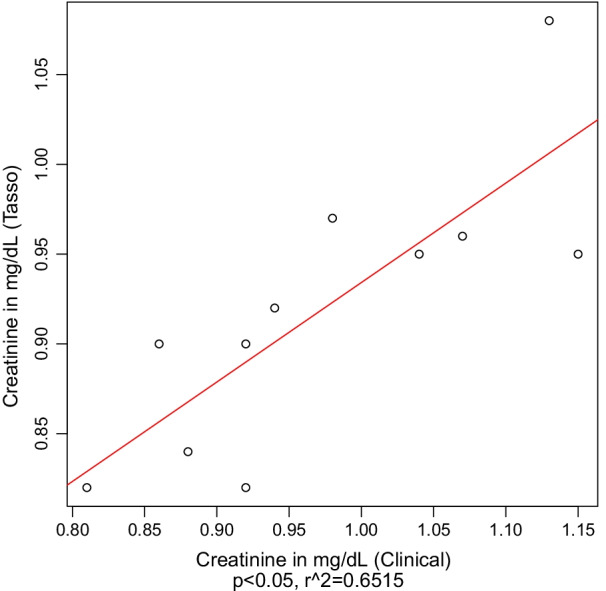
Comparison of creatinine values from self-collected Tasso vs. clinical venipuncture specimens

Serum creatinine results from clinician-collected venipuncture specimens yielded slightly higher values than self-collected specimens, with a mean difference of + 0.05 mg/dL (95% confidence interval: 0.014–0.093). Figure 2 depicts the overall positive correlation between paired samples (r^2^ = 0.65, *P* = 0.007), with venipuncture specimens yielding slightly higher results compared to Tasso specimens (Additional file 6: Table S3). Overall, 10 of 11 values fell within our pre-specified range of agreement.

Among participants who attempted blood collection using only one Tasso device, 5 of 21 (24%) samples were insufficient to complete any testing. Of the 27 participants who used two devices, 27 (100%) had sufficient specimen to perform at least one test and 24 (89%) collected enough specimen to perform two tests (Table 3). Venipuncture results for HIV, creatinine and RPR were available for 21 participants; 9 (43%) of these 21 participants self-collected sufficient serum using two devices to perform all three tests.

**Table 3:** Proportion of PrEP Monitoring Samples Successfully Tested by Number of Self-Collection Attempts

Number of collections attempted using Tasso device	PrEP monitoring tests completed^a^			
	0	1	2	3
1 (n = 21)	6 (28.6%)^b^	11 (52.4%)	4 (19.0%)	0
2 (n = 27)	0	3 (11.1%)	15 (55.6%)	9 (33.3%)
Overall (n = 48)	6 (12.5%)	14 (29.2)	19 (39.6)	9 (18.8%)

^a^Tests included serum HIV antigen–antibody, rapid plasma reagin and creatinine

^b^Of 5 participants with insufficient sample to complete at least one PrEP lab test: 3 reported methamphetamine or heavy alcohol use in the last 8 h, 1 reported history of “quick clotting,” 1 insufficient volume after centrifugation. One specimen could not be analyzed due to a laboratory processing error

Overall, 45 (94%) of 48 participants did not find the blood collection process difficult and 37 (95%) of 39 felt they could do self-collection without supervision at home. Forty-five (94%) participants expressed no major reservations about self-collecting samples at home. The three possible concerns with HB-PrEP identified were wait time during blood collection, stability of blood samples, and the potential for samples to be lost in the mail.

## Discussion

As we work toward US HIV prevention goals [18], the number of PrEP users is expected to increase. New strategies to expand the healthcare system’s capacity to provide PrEP and ensure that users continue to receive appropriate monitoring and STI screening will be essential. Our pilot study found that interest in HB-PrEP services was high among current and prospective PrEP users, that self-collection of blood specimens using the Tasso device was acceptable and feasible, and that the results of HIV antigen/antibody EIA, quantitative RPR, and creatinine testing from specimens collected using this method showed good agreement with routinely collected venipuncture samples.

Nearly 45% of study participants reported a prior history of syphilis and 12% were serofast (stable and persistent positive RPR titer after treatment; data not shown), highlighting the importance of performing quantitative nontreponemal serologic testing to identify new infections among PrEP patients with a history of syphilis. While several PrEP delivery models exist that facilitate PrEP monitoring outside of traditional clinics [19], few have been designed to allow PrEP users to collect samples entirely from home for laboratory-based 4th generation HIV and quantitative syphilis testing. Participants in the PrEPTECH study were mailed kits with extragenital gonorrhea and chlamydia swabs to collect at home but were required to present to a local laboratory to have blood drawn [5]. In the PrEP@Home and the more recent Iowa TelePrEP studies, participants were able to self-collect all PrEP monitoring samples from home; however, whole blood was collected by fingerstick for RPR testing at a fixed titer and for HIV using a rapid antibody assay [7, 8]. Our findings demonstrate that the Tasso device allows for easy blood specimen collection, and that serum samples collected with the device can be used to perform quantitative RPR titers and laboratory HIV antigen/antibody testing.

While we believe that our findings are encouraging, one Tasso device did not allow participants to collect enough blood for PrEP monitoring. Even the use of two devices resulted in insufficient specimen to perform two tests in 11% of participants and insufficient specimen to perform all three monitoring tests in 67% of participants. A newer Tasso device with increased capacity to collect 500–1000 µl—2–3 times the quantity collected by the current device—is currently in production and may solve this problem. It may also be possible to increase sample collection by improving local blood flow with the use of heating packs.

Our finding that most participants preferred home-based sampling over coming into clinic is consistent with conclusions from three other studies, [5, 7, 20] but whether these reported preferences will translate to consistent use of kits for HB-PrEP is uncertain. Chasco, et al. found that the majority of individuals who were offered a home monitoring option did not prefer self-collection of blood and that use of home kits was associated with lower odds of completing HIV, syphilis and creatinine testing compared to standard laboratory collections [8]. Our preliminary data demonstrating high interest in HB-PrEP and the acceptability and feasibility of blood specimen self-collection using the Tasso device do not prove that HB-PrEP using the Tasso device will be successful. We are currently implementing a larger scale, hybrid randomized controlled trial (HOT4PrEP: Home Option Testing for PrEP) to examine the feasibility of blood specimen self-collection using the newer, higher capacity Tasso device in the home setting and to evaluate the real-world effectiveness of home kits on PrEP retention.

We acknowledge several limitations of this study. A low proportion of patients offered enrollment in our online survey participated in the study and our findings may be subject to selection bias. Consents, videos and surveys were only available in English, and our findings may not be generalizable to non-English speakers. Although a high proportion (84%) of persons offered enrollment in our study of self-collection participated, the population enrolled was a convenience sample of uncertain external generalizability. Our study comparing self-collected to venipuncture specimens included a small number of specimens which limited our ability to compare results. While we attempted to create conditions that might parallel those for collection in the home setting (i.e., participants collecting blood samples alone and without direct supervision), we recognize that the study took place in a supported environment where staff was available as needed for assistance. Also, as samples were not mailed in from home, we were unable to evaluate the effect of time from collection to processing or sample storage conditions on testing results in this study. However, samples self-collected using the Tasso device that were temperature-stressed and processed 72 h after collection yielded valid serologic results for SARS-CoV-2 antibodies [12]. Our upcoming hybrid randomized controlled trial will address several of these limitations by enrolling a larger and more diverse sample of participants and evaluate how often patients actually use the Tasso device and the impact of a home-based, self-collection option on PrEP retention.

## Conclusions

In conclusion, among a study population attending an urban SHC we found use of a self-collection device for home PrEP monitoring was acceptable and preferred by PrEP patients, that it was feasible for patients to self-collect specimens with minimal instruction, and that test results from self-collected specimens demonstrated high agreement with clinician-collected venipuncture specimens. Our study also suggests that home self-collection of blood specimens using the Tasso device could overcome one of the primary limitations of existing HB-PrEP programs, the inability to collect serum for quantitative RPR and 4th generation HIV testing. While this pre-implementation pilot study only evaluated the feasibility and acceptability of blood sample self-collection and general interest in HB-PrEP, use of the Tasso device as a key component of a larger HB-PrEP program could have the potential to help meet the increasing need for STI screening and monitoring of PrEP patients during the COVID-19 pandemic and beyond. Despite these encouraging findings, further work is needed to refine blood self-collection procedures to ensure consistent and adequate specimen volumes for PrEP monitoring tests and to definitively evaluate our proposed approach to HB-PrEP.

## Supplementary Information

**Additional file 1**: “HOT4PrEP Acceptability Survey (Online)”. Survey instrument used to collect responses from online participants.

**Additional file 2**: “HOT4PrEP Acceptability Survey (In Person)”. Survey instrument used to collect responses from in-person participants.

**Additional file 3**: “Tasso device self-collection instructions”. Visual instruction sheet provided to participants who self-collected blood samples. Note: The original instruction sheet was sourced from Tasso, Inc. and modified for use in the study. Study staff were provided written authorization to use the sheet with minor modifications.

**Additional file 4**: **Table S1**. Results of HIV antigen–antibody EIA for paired self-collected vs. standard venipuncture samples. Counts and sums of HIV results by collection method.

**Additional file 5**: **Table S2**. Results of qualitative RPR for paired self-collected vs. standard venipuncture samples. Counts and sums of qualitative RPR results by collection method.

**Additional file 6**: **Table S3**. Comparison of serum creatinine results from paired venipuncture and self-collected samples. Exact values with summary statistics of paired creatinine sample results.

## Data Availability

Exact counts and values for HIV, creatinine and RPR results are included in the main text or as Additional files. Transcripts of additional comments from participants provided in surveys are not included as responses may be identifiable. Otherwise, the datasets used and/or analyzed during the study are available from the corresponding author on reasonable request.

## Abbreviations

PrEP: Pre-exposure prophylaxis
STI: Sexually transmitted infections
RPR: Rapid plasma reagin
HB-PrEP: Home-based PrEP
PHSKC: Public Health-Seattle & King County
SHC: Sexual health clinic
EIA: Enzyme-linked immunosorbent assay
PPA: Positive percent agreement
NPA: Negative percent agreement
