# Supplementary material for: Feasibility of a novel self-collection method for blood samples and its acceptability for future home-based PrEP monitoring
Source: BMC Infect Dis. 2022 May 13;22:459. doi: 10.1186/s12879-022-07432-0 (PMC9100305; doi:10.1186/s12879-022-07432-0)
Supplement: Supplementary file 1 — Additional file 1: “HOT4PrEP Acceptability Survey (Online)”. Survey instrument used to collect responses from online participants. [file 12879_2022_7432_MOESM1_ESM.pdf]

HOT4PrEP: Acceptability Survey (Online)

Thank you for participating in our study! Your input is very important for us to understand how we can best create an option to decrease the number of times you need to come in to clinic and help you stay on PrEP from home. We are particularly interested in your thoughts now during COVID-19 "stay at home" restrictions and also your preferences for PrEP care after the epidemic ends.

If you chose to do home testing, we would send you a kit containing swabs to do gonorrhea/chlamydia testing from the mouth and rectum (bottom), as well as supplies to collect your own blood samples from the upper arm that we would use to test HIV, syphilis and kidney function. You would then send the kit back to us in the mail using the included shipping label.

Please answer the questions below about this home-based option. You can skip any questions you wish or save your progress to come back later. If you choose to be contacted in the future, name and contact info will be required.

Age (years)	______________________
Sex assigned at birth	◯ Male at birth<br>◯ Female at birth<br>◯ Intersex
Current gender identity	◯ Cisgender man<br>◯ Transgender woman<br>◯ Non-binary<br>◯ Agender<br>◯ Other not listed above
Race/ethnicity	◯ Non-Hispanic Black, African-American<br>◯ Non-Hispanic White<br>◯ Latinx<br>◯ Asian<br>◯ Native American/First Nation<br>◯ Mixed race<br>◯ Other not listed above
Gender of sex partners<br>(Check all that apply.)	☐ Cisgender men<br>☐ Cisgender women<br>☐ Transgender men<br>☐ Transgender women<br>☐ Agender/non-binary<br>☐ Other not listed above
Have you ever used PrEP before?	◯ Yes<br>◯ Not yet
Approximately how long have you been using PrEP?<br>(years)	______________________
Given the current restrictions on in-person PrEP care due to the COVID-19 epidemic, would you be interested in an option to collect your own samples from home?	◯ Yes<br>◯ No<br>◯ Maybe<br>◯ Not Sure

If you had the option to do PrEP labs from home in the future once COVID-19 restrictions end, would you prefer to do this over coming into clinic?	◯ Yes<br>◯ No<br>◯ Maybe<br>◯ Not Sure
People on PrEP usually have HIV/STI testing 4 times per year. One visit per year will need to be in person. How often would you want to collect home test samples?	◯ Once per year<br>◯ 2x per year<br>◯ 3x per year
Would you prefer to receive several kits in advance with text/email reminders when to submit them or receive one kit at a time before testing is due?	◯ Several kits in advance<br>◯ One kit at a time
How would you prefer to be reminded when to send in a kit? (Check any/all that apply.)	☐ Telephone call<br>☐ Text message reminder<br>☐ Email reminder<br>☐ MyChart reminder
For the times you mail in kits, would you be interested in speaking with a provider by phone, video or text as well?	◯ Yes<br>◯ No<br>◯ Maybe<br>◯ Not Sure
What method of communication would you prefer with the provider? (Check any/all that apply.)	☐ Telephone call<br>☐ Video chat<br>☐ Text message check-in<br>☐ Email check-in
Would the option to mail kits in from home make you more likely to stay on PrEP?	◯ More likely<br>◯ Less likely<br>◯ About the same<br>◯ I don't know
Recall the "Hemolink"/Tasso device video you watched. Do you think you could collect blood from the upper arm into a small tube like this at home?	◯ Yes<br>◯ No<br>◯ Maybe<br>◯ Not Sure
Would you prefer to collect your own blood samples from the upper arm using the "Hemolink"/Tasso device or have a nurse draw your blood in clinic?	◯ I would prefer to collect my own sample<br>◯ I would prefer someone else draw my blood<br>◯ Not sure or I don't know<br>◯ I don't have a preference
What are your concerns, if any, about collecting your own samples at home?	______________________________
If one of your home tests were abnormal or positive, we would contact you directly to discuss the results. If all of your tests were normal or negative, how would you prefer to find out results? (Check all that apply.)	☐ MyChart message<br>☐ Weltel message<br>☐ Secure video conference on Zoom<br>☐ I would call in to private clinic phone line for results<br>☐ I would like someone to call me with results<br>☐ Come in to clinic to receive results

Once we can return to in-person appointments, we are considering having a nurse do visits with patients when they come to clinic rather than meeting with the PrEP coordinators . Who would you "prefer to see" in clinic during future PrEP visits?	◯ A nurse practitioner (NP) or physician assistant (PA)<br>◯ A nurse (RN)<br>◯ One of the PrEP coordinators<br>◯ Both a nurse and a PrEP coordinator<br>◯ Both a NP/PA and a PrEP coordinator<br>◯ Doesn't matter to me
Please provide any additional ideas or comments on how we can make PrEP services from home better for our patients.	__________________________________
Would you like us to keep your name and phone number or email address to contact you if we decide to offer home testing in the future?	◯ Yes<br>◯ No
Name	______________________
Phone number	______________________
Email address	______________________
