# Supplementary material for: Feasibility of a novel self-collection method for blood samples and its acceptability for future home-based PrEP monitoring
Source: BMC Infect Dis. 2022 May 13;22:459. doi: 10.1186/s12879-022-07432-0 (PMC9100305; doi:10.1186/s12879-022-07432-0)
Supplement: Supplementary file 2 — Additional file 2: “HOT4PrEP Acceptability Survey (In Person)”. Survey instrument used to collect responses from in-person participants. [file 12879_2022_7432_MOESM2_ESM.pdf]

HOT4PrEP: Acceptability Survey (In Person)

In what portion of the study has the participant elected to participate?	◯ PrEP-eligible/using: Survey only<br>◯ PrEP-eligible/using: Survey + blood collection<br>◯ HIV-positive: blood collection only<br>◯ Syphilis known/suspected: blood collection only<br>◯ Syphilis known/suspected and PrEP-eligible/using: Survey + blood collection
View instructional video below before proceeding with blood collection.<br><br>Follow this link for fullscreen version: https://www.tassoinc.com/tasso-sst-video	
Open instruction sheet here.<br><br>[Attachment: "TASSO-LH IFU, IUO.pdf"]	
Age (years)	__________________________
Sex assigned at birth	◯ Male at birth<br>◯ Female at birth<br>◯ Intersex
Current gender identity	◯ Cisgender man<br>◯ Cisgender woman<br>◯ Transgender man<br>◯ Transgender woman<br>◯ Non-binary<br>◯ Agender<br>◯ Other not listed above
Race/ethnicity	◯ Non-Hispanic Black/African-American<br>◯ Non-Hispanic White<br>◯ Latinx<br>◯ Asian<br>◯ Native American/First Nation<br>◯ Mixed race<br>◯ Other not listed above
List your race/ethnicity.	__________________________
Gender of sex partners	☐ Cisgender men<br>☐ Cisgender women<br>☐ Transgender men<br>☐ Transgender women<br>☐ Agender/non-binary<br>☐ Other not listed above
Have you started PrEP yet (used ever before)?	◯ Yes<br>◯ No
Approximately how long have you been using PrEP? (years)	__________________________

We are considering having a nurse do visits with patients when they come to clinic rather than meeting with the PrEP coordinators . Who would you "prefer to see" in clinic during future PrEP visits?

- ◯ A nurse practitioner (NP) or physician assistant (PA)
- ◯ A nurse (RN)
- ◯ One of the PrEP coordinators
- ◯ Both a nurse and a PrEP coordinator
- ◯ Both a NP/PA and a PrEP coordinator
- ◯ Doesn't matter to me

If the option to do your labs from home were available, would you prefer to do this over coming into clinic?

- ◯ Yes
- ◯ No
- ◯ Maybe
- ◯ Not Sure

How do restrictions due to COVID-19 affect your interest in a home testing option?

- ◯ I am interested in a home option only during the COVID epidemic
- ◯ I am interested in a home option only after the COVID epidemic ends
- ◯ I would be interested in a home option both now and after COVID the epidemic ends
- ◯ I'm not sure

If home testing required you to collect blood into a small tube, would you prefer to collect specimens at home or come to the clinic?

- ◯ Collect blood sample at home
- ◯ Come to clinic for blood draw

People on PrEP usually have HIV/STI testing 4 times per year. One visit per year will need to be in person. How often would you want to collect home test samples?

- ◯ Once per year
- ◯ 2x per year
- ◯ 3x per year

Would you prefer to receive kits in advance with text/email reminders when to submit them or receive one kit at a time every 3 months in the mail?

- ◯ Kits in advance with reminders
- ◯ One kit in mail every 3 months

How would you prefer to be reminded when to send in a kit? (Check any/all that apply.)

- ☐ Telephone call
- ☐ Text message reminder
- ☐ Email reminder
- ☐ MyChart reminder

For the times you mail in kits, would you be interested in speaking with a provider by phone, video or text as well?

- ◯ Yes
- ◯ No
- ◯ Maybe
- ◯ Not Sure

What method of communication would you prefer with the provider? (Check any/all that apply.)

- ☐ Telephone call
- ☐ Video chat
- ☐ Text message check-in
- ☐ Email check-in

If one of your home tests were abnormal or positive, we would contact you directly to discuss the results. If all of your tests were normal or negative, how would you prefer to find out results? (Check all that apply.)

- ☐ MyChart message
- ☐ Weltel message
- ☐ Secure video conference on Zoom
- ☐ I would call in to private clinic phone line for results
- ☐ I would like someone to call me with results
- ☐ Come in to clinic to receive results

Would the option to mail kits in from home make you more likely to stay on PrEP?	◯ More likely<br>◯ Less likely<br>◯ About the same<br>◯ I don't know
Did you feel the video explained clearly how to collect your blood sample at home?	◯ Yes<br>◯ No<br>◯ Somewhat<br>◯ Not Sure
Did you feel the instruction sheet explained clearly how to collect your blood sample at home?	◯ Yes<br>◯ No<br>◯ Somewhat<br>◯ Not Sure
Was there something you wished the video or instruction sheet had covered that they didn't?	__________________________
Did you find the process to collect blood was difficult?	◯ Yes<br>◯ No<br>◯ Somewhat<br>◯ Not Sure
Do you think it was helpful to do the blood collection with help the first time in clinic?	◯ Yes<br>◯ No<br>◯ Somewhat<br>◯ Not Sure
Do you feel you could collect a blood sample like this on your own at home?	◯ Yes<br>◯ No<br>◯ Maybe<br>◯ Not Sure
What are your concerns, if any, about collecting a blood sample at home?	________________________________
Have you ever had syphilis before?	◯ Yes<br>◯ No<br>◯ Not Sure
If we could not perform syphilis testing on samples from the self-collection kits, would you still be interested in receiving kits at home that could test for HIV, gonorrhea, chlamydia and kidney function only?	◯ Yes<br>◯ No<br>◯ Maybe<br>◯ Not Sure
Please provide any additional ideas or comments on how we can make PrEP services from home better for our patients.	________________________________
Would you like us to keep your name and phone number or email address to contact you if we decide to offer home testing in the future?	◯ Yes<br>◯ No
Name	__________________________

Phone number

____

E-mail address

____
