# Supplementary material for: Feasibility of a novel self-collection method for blood samples and its acceptability for future home-based PrEP monitoring
Source: BMC Infect Dis. 2022 May 13;22:459. doi: 10.1186/s12879-022-07432-0 (PMC9100305; doi:10.1186/s12879-022-07432-0)
Supplement: Supplementary file 3 — Additional file 3: “Tasso device self-collection instructions”. Visual instruction sheet provided to participants who self-collected blood samples. Note: The original instruction sheet was sourced from Tasso, Inc. and modified for use in the study. Study staff were provided written authorization to use the sheet with minor modifications. [file 12879_2022_7432_MOESM3_ESM.pdf]

TASSO-LH

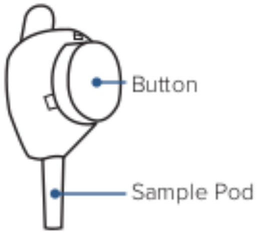

INCLUDED

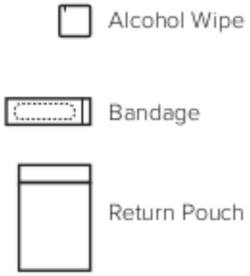

PREPARE

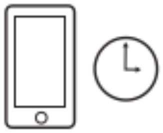

1. GET TIMER

You will start a three minute timer in step 9.

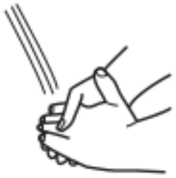

2. WASH HANDS

Use soap and warm water.

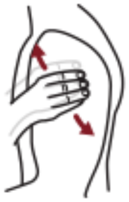

3. WARM ARM

Expose shoulder and rub quickly just below the shoulder until it feels warm.

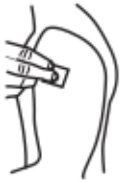

4. CLEAN ARM

Using the alcohol pad, wipe the warmed area. Let it dry.

COLLECT

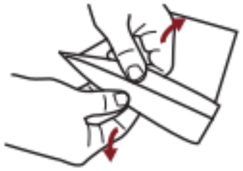

5. OPEN POUCH

Pull the two layers of the device pouch apart from the middle to open.

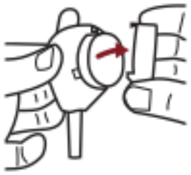

6. REMOVE CAP

Pull off the clear plastic cover over the red button.

DO NOT PRESS THE RED BUTTON YET.

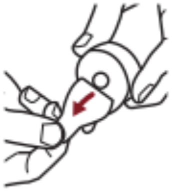

7. PEEL BACKING

Pull the tab behind the red button to remove the paper backing.

KEEP SAMPLE POD POINTING DOWN.

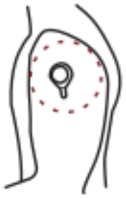

8. STICK DEVICE

Stick the device to the warm and clean area on shoulder.

DO NOT REMOVE ONCE IT'S ON.

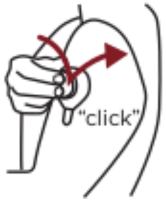

9. PRESS BUTTON

Press the red button firmly. Let go when you hear a loud click. You won't see blood right away.

START A 3 MINUTE TIMER.

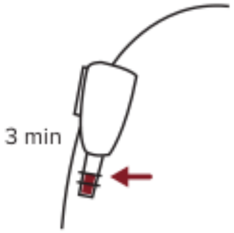

10. WAIT FOR BLOOD

Allow tube to fill to the top (no space visible at the top of the tube) before going to next step.

KEEP ARM AT SIDE DURING COLLECTION.

RETURN

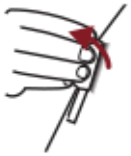

11. PEEL OFF DEVICE

Slowly peel the device off arm.

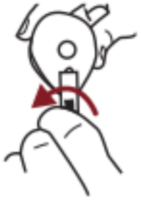

12. REMOVE TUBE

Remove tube by firmly twisting it a quarter turn and pulling down.

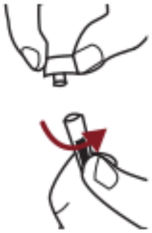

13. CAP TUBE

Twist the cap firmly onto the tube.

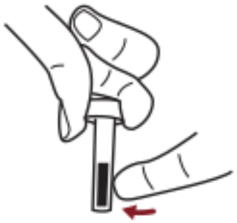

14. MIX BLOOD

Flick the side of the tube a couple of times to mix the blood.

DO NOT SHAKE.

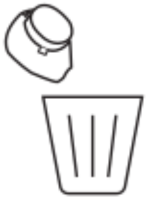

15. DISCARD BUTTON

Used device may be placed in the sharps container or standard trash.
