# Supplementary material for: Feasibility of a novel self-collection method for blood samples and its acceptability for future home-based PrEP monitoring
Source: BMC Infect Dis. 2022 May 13;22:459. doi: 10.1186/s12879-022-07432-0 (PMC9100305; doi:10.1186/s12879-022-07432-0)
Supplement: Supplementary file 4 — Additional file 4: Table S1. Results of HIV antigen–antibody EIA for paired self-collected vs. standard venipuncture samples. Counts and sums of HIV results by collection method. [file 12879_2022_7432_MOESM4_ESM.docx]

| **Table S1: Results of HIV antigen-antibody EIA for paired self-collected vs standard clinical venipuncture samples** | | | |
| --- | --- | --- | --- |
|  | **Venipuncture sample result** | |  |
|  | *Negative* | *Positive* | ***Total*** |
| **Self-collected sample result** |  | |  |
| *Negative* | 29 | 0 | 29 |
| *Positive* | 0 | 7 | 7 |
| *QNS** | 6 | 3 | 9 |
| ***Total*** | 35 | 10 | 46 |

For 36 specimens tested, PPA (percent positive agreement) = 7/7 (100%) and NPA (negative percent agreement) is 29/29 (100%).

*Quantity not sufficient: 4 were unable to collect specimen on either attempt; 5 cases had insufficient sample remaining for HIV testing after completing syphilis and/or creatinine testing
