# Supplementary material for: Feasibility of a novel self-collection method for blood samples and its acceptability for future home-based PrEP monitoring
Source: BMC Infect Dis. 2022 May 13;22:459. doi: 10.1186/s12879-022-07432-0 (PMC9100305; doi:10.1186/s12879-022-07432-0)
Supplement: Supplementary file 5 — Additional file 5: Table S2. Results of qualitative RPR for paired self-collected vs. standard venipuncture samples. Counts and sums of qualitative RPR results by collection method. [file 12879_2022_7432_MOESM5_ESM.docx]

| **Table S2: Results of qualitative RPR for paired self-collected vs standard venipuncture samples** | | | |
| --- | --- | --- | --- |
|  | **Venipuncture sample result** | |  |
|  | *Nonreactive* | *Reactive* | ***Total*** |
| **Self-collected sample result** |  | |  |
| *Nonreactive* | 20 | 1^ | 21 |
| *Reactive* | 0 | 10 | 10 |
| *QNS** | 6 | 7 | 13 |
| ***Total*** | 26 | 19 | 45 |

For 31 tested specimens, PPA (percent positive agreement) = 10/10 (100%) and NPA (negative percent agreement) is 20/21 (95.2%).

^T. pallidum antibody EIA performed on clinical sample was negative

*Quantity not sufficient: 3 were unable to collect specimen on either attempt; 10 cases had insufficient sample remaining for syphilis testing after HIV and/or creatinine testing
