# Supplementary material for: Feasibility of a novel self-collection method for blood samples and its acceptability for future home-based PrEP monitoring
Source: BMC Infect Dis. 2022 May 13;22:459. doi: 10.1186/s12879-022-07432-0 (PMC9100305; doi:10.1186/s12879-022-07432-0)
Supplement: Supplementary file 6 — Additional file 6: Table S3. Comparison of serum creatinine results from paired venipuncture and self-collected samples. Exact values with summary statistics of paired creatinine sample results. [file 12879_2022_7432_MOESM6_ESM.docx]

| **Table S3: Comparison of serum creatinine results from paired venipuncture and self-collected samples** | | | |
| --- | --- | --- | --- |
| **Participant** | **Creatinine (mg/dL), venipuncture sample** | **Creatinine (mg/dL), Tasso sample** | **Difference in creatinine samples**  **(Venipuncture – Tasso), mg/dL** |
| **A** | **0.98** | 0.97 | 0.01 |
| **B** | **0.92** | 0.82 | 0.10 |
| **C** | **1.07** | 0.96 | 0.11 |
| **D** | **0.81** | 0.82 | -0.01 |
| **E** | **1.15** | 0.95 | 0.20 |
| **F** | **0.92** | 0.9 | 0.02 |
| **G** | **1.13** | 1.08 | 0.05 |
| **H** | **0.88** | 0.84 | 0.04 |
| **I** | **1.04** | 0.95 | 0.09 |
| **J** | **0.94** | 0.92 | 0.02 |
| **K** | **0.86** | 0.90 | -0.04 |
| Mean | 0.97 | 0.92 | 0.05 |
| Standard deviation | 0.11 | 0.08 | 0.07 |
